# lncRNA-KCNQ1OT1: A Potential Target in Exosomes Derived from Adipose-Derived Stem Cells for the Treatment of Osteoporosis

**DOI:** 10.1155/2021/7690006

**Published:** 2021-10-19

**Authors:** Shan-zheng Wang, Jun Jia, Chang-hong Chen

**Affiliations:** ^1^Department of Orthopaedics, Zhongda Hospital, Medical School of Southeast University, 87 Ding Jia Qiao Road, Nanjing, Jiangsu 210009, China; ^2^Department of Orthopaedics, The 904th Hospital of Joint Logistic Support Force, PLA, 101 Xingyuan North Road, Wuxi, Jiangsu 214000, China; ^3^Department of Orthopaedics, Jiangyin Hospital Affiliated to Nanjing University of Chinese Medicine, 130 Renmin Middle Road, Jiangyin, Jiangsu 214400, China

## Abstract

**Background:**

Osteoporosis is a worldwide medical and socioeconomic burden characterized by systemic impairment of bone strength and microstructure. Exosomes derived from adipose-derived stem cells (ADSCs-Exos) have been confirmed to play effective roles in the repair of various tissues and organs. This study was aimed at investigating the role of ADSCs-Exos and a novel long noncoding RNA KCNQ1OT1 played in osteoporosis as well as the underlying mechanism.

**Methods:**

Primary osteoblasts were treated with different doses of tumor necrosis factor-*α* (TNF-*α*) (0, 1, 2.5, 5, and 10 ng/ml) and then cocultured with ADSCs-Exos or exosome-derived from lnc-KCNQ1OT1-modified ADSCs (KCNQ1OT1-Exos). The expression of miRNA-141-5p (miR-141-5p) and lnc-KCNQ1OT1 was evaluated by quantitative real-time polymerase chain reaction (qRT-PCR). The protein expression of cleaved-caspase-3, caspase-3, and Bax was determined by Western blot. Cell viability and apoptosis were assessed by Cell Counting Kit-8 (CCK-8) and flow cytometry analysis, respectively. The binding sites between KCNQ1OT1 and miR-141-5p were validated by dual-luciferase reporter assay.

**Results:**

TNF-*α* dose-dependently increased miR-141-5p expression, inhibited viability, and promoted apoptosis of osteoblasts. However, miR-141-5p silencing or cocultured with ADSCs-Exos attenuated these effects. In addition, KCNQ1OT1-Exos could more significantly attenuate the induced cytotoxicity and apoptosis compared to ADSCs-Exos. Moreover, miR-141-5p was confirmed as the target of KCNQ1OT1 by luciferase reporter assay.

**Conclusions:**

ADSCs-Exos can attenuate cytotoxicity and apoptosis of TNF-*α*-induced primary osteoblasts. KCNQ1OT1-Exos have a more significant inhibitory effect compared to ADSCs-Exos by the function of sponging miR-141-5p, suggesting that KCNQ1OT1-Exos can be promising agents in osteoporosis treatment.

## 1. Introduction

Osteoporosis is a common skeletal disease characterized by structural disorders of bone mass caused by increased osteoclast activity and reduced osteoblast generation [1]. Clinical treatment strategies are often based on the promotion of osteoblast proliferation and osteoclast inhibition [2, 3]. In osteoporosis pathogenesis, tumor necrosis factor-*α* (TNF-*α*) is a proinflammatory cytokine which has been revealed to contribute to osteoporosis by regulating both osteoblasts and osteoclasts [3, 4]. TNF-*α* can suppress bone formation and inhibit osteoblast differentiation by inducing cytotoxicity and apoptosis [5, 6]. Therefore, exploring new pathways to arrest TNF-*α*-induced cytotoxicity and apoptosis in osteoporosis is necessary.

As a subset of mesenchymal stem cells (MSCs), adipose-derived stem cells (ADSCs) can be easily obtained from adipose tissues and possess the potential of multidifferentiation and self-renewal [7, 8]. ADSCs are found in abundant quantities and can be easily obtained by minimally invasive procedures [9]. Autologous ADSCs can be used to promote bone regeneration under osteoporotic conditions [10]. Thus, the application of ADSCs might be a promising MSC-based strategy for bone formation and structural remodeling in osteoporosis [11, 12].

Exosomes are small, membrane-bound extracellular vesicles that are enriched in selected proteins, lipids, nucleic acids, and glycoconjugates [13]. Exosomes have been proved to play a significant role in the etiology of bone metabolic diseases, especially osteoporosis [14]. Exosomes derived from ADSCs (ADSCs-Exos) contain bioactive substances of ADSCs and might play a similar role to ADSCs [15]. Recent researches have demonstrated that ADSCs-Exos had stimulating effects in the repair of a variety of tissues and organs [10, 16, 17]. Nevertheless, the potential therapeutic effects of ADSCs-Exos on osteoporosis and the underlying mechanism need further investigations.

Exosomes are important mediators between cells by transferring molecules, such as long noncoding RNAs (lncRNA), microRNAs (miRNAs), and cytokines [18–21]. Evidence indicates that exosomes derived from mmu_circ_0000250-modified ADSCs are able to promote wound healing in diabetic mice [22]. Additionally, exosomes derived from miR-188-3p-modified ADSCs can protect Parkinson's disease [23]. Recently, KCNQ1overlapping transcript 1 (lnc-KCNQ1OT1) was found to be able to positively regulate osteogenic differentiation of bone marrow mesenchymal stem cells (BMSCs) by acting as a ceRNA to regulate BMP2 expression through sponging miR-214 [24]. However, few studies have investigated the potential role of KCNQ1OT1 in the treatment of osteoporosis. This study was aimed at investigating the role of ADSCs-Exos and KCNQ1OT1 played in osteoporosis as well as the underlying mechanism.

## 2. Methods

### 2.1. Isolation and Culture of ADSCs

The Institutional Animal Care and Use Committee of Southeast University approved the protocol for the use of animals in this study. Adipose tissues were collected from the inguinal fat pad of 2-3-week-old C57BL/6 mice (SLAC, Shanghai, China) and then rinsed in phosphate-buffered saline (PBS) and cut into 1 × 1 mm pieces. The collected tissues were digested by collagenase type II (Sigma-Aldrich, USA) at 37°C for 1 h. After digestion, tissues were centrifuged at room temperature (1000 rpm, 5 min) and the resultant cell pellet was resuspended in Dulbecco's modified Eagle's medium (DMEM) at the cell density of 5 × 10^6^/ml. Cells were then cultured at 37°C under a 5% CO_2_ atmosphere. The culture medium was replaced every 2-3 days, and cells after 3 passages were used in the present study.

### 2.2. Characterization of Isolated ADSCs

ADSCs were cultured in a 24-well plate at a density of 4 × 10^4^ cells/well with basic culture medium (DMEM-LG) containing 10% FBS, 100 U/ml penicillin, and 100 mg/ml streptomycin. They were subjected to induced differentiation by culturing them in osteogenic (Cyagen Biosciences, Rasmx-90021), adipogenic (Cyagen Biosciences, Rasmx-90031), and chondrogenic (Cyagen Biosciences, Rasmx-90041) medium, respectively. The outcomes were evaluated by Alizarin Red, Oil Red O, and Alcian Blue staining, respectively. In addition, MSC surface markers (CD90^+^ and CD34-) were detected by flow cytometric analysis. Briefly, adherent cells from passage 4 were resuspended and then incubated in FACS buffer containing 2 *μ*l of Fc receptor-blocking reagent (FcX Blocker, BioLegend, San Diego, CA, USA). Cells were stained with reagent to exclude dead cells (Zombie NIR, Japan). After washing the cells, IC fixation buffer (Affymetrix, Japan) was added. Permeabilization buffer (Affymetrix, Japan) was diluted with purified water for 10 times. After permeabilization, CD90 and CD34 antibodies were used to stain the cells. All antibodies were from BD Biosciences (Tokyo, Japan). Stained cells were sorted by flow cytometry (Accuri C6, BD), and data was analyzed with FlowJo software (Tree Star, Ashland, OR, USA).

### 2.3. Isolation and Characterization of Primary Osteoblasts

The isolation and characterization of primary osteoblasts were conducted as described before [25]. Briefly, calvarium tissues from C57BL/6 mice were collected and digested in 0.25% trypsin (Thermo Fisher, USA) containing 0.02% EDTA for 25 min at 37°C. Then, the tissues were digested in 5 ml Hanks solution containing 0.1% collagenase I (Thermo Fisher, USA) and 0.05% trypsin for 1 h in a shaking incubator at 37°C with a shaking speed of 200 r/min. The released cells were collected by centrifugation for 10 minutes at 1500 r/min. The cells were suspended in 5 ml of a-MEM (containing 1 g/L D-Glucose and L-Glutamine, BI, USA) containing 10% FBS and then transferred to 25 cm^2^ plastic culture flasks (Nest, China). The culture flasks were incubated at 37°C in a 5% CO_2_ incubator. The images of cell morphology were taken using a microscope attaching camera (OLYMPUS, IX51). The primary osteoblasts of passage 4 were prepared for further experiments and characterized by alkaline phosphatase staining and Alizarin Red S staining.

### 2.4. KCNQ1OT1 Transfection

To overexpress lncRNA-KCNQ1OT1, the lncRNA-KCNQ1OT1 mimic was subcloned into pcDNA-3.1 vector (Invitrogen, USA) to generate pcDNA-lnc-KCNQ1OT1 constructs. For stable transduction of pcDNA-lnc-KCNQ1OT1 or pcDNA-3.1 (negative control, NC), pcDNA-lnc-KCNQ1OT1 or NC was transfected into ADSCs using Lipofectamine 2000 (Invitrogen, USA) according to the manufacturer's instructions.

### 2.5. Isolation and Characterization of Exosomes

At 48 h posttransfection, a Total Exosome Isolation kit (Invitrogen, USA) was applied to isolate the total exosomes from the supernatant of ADSC culture medium and culture medium transfected with pcDNA-lnc-KCNQ1OT1 or NC (ADSCs-Exos, LV-KCNQ1OT1-Exos, or LV-NC-Exos, respectively) according to the manufacturer's protocol [26]. Bicinchoninic acid (BCA) protein assay kit (MACGENE, China) was used to measure the concentration of isolated exosomes. Before use, all exosome samples were analyzed for proper size by nanoparticle tracking analysis (NTA; NanoSight, Malvern) and for morphology by transmission electron microscopy (TEM). The protein levels of CD9, CD63, CD81, and Alix (representative markers of exosomes) were then detected.

### 2.6. Exosome Uptake Assay

ADSCs-Exos were labeled with PKH26 (Sigma Aldrich, USA) according to the manufacturer's protocol. Isolated exosomes were resuspended in diluent C (1 ml). Then, 6 *μ*l PKH26 was added. ADSCs-Exos and PKH26 solutions were mixed for 30 s and then centrifuged (120000 × g, 2 h, 4°C). Exosomes were resuspended in the complete culture medium, and the PKH26-labeled ADSC-Exo solution was added into primary osteoblasts for incubation. After 24 h of culture, osteoblast cells were fixed with 4% formaldehyde for 10 min. DAPI was used to stain the nuclei.

### 2.7. Cell Viability Assay

The viability of primary osteoblasts was determined by the Cell Counting Kit-8 (CCK-8; Dojindo, Japan) assay. Briefly, cells were seeded into 96-well plates (2 × 10^4^ cell/ml) and incubated for 24 h. Then, 10 *μ*l CCK-8 solution was added into each well for incubation of 1-2 h (37°C, 5% CO_2_). Subsequently, the optical density (OD) value was measured at 450 nm using a spectrophotometer (Bio-Rad, USA).

### 2.8. Cell Apoptosis Assay

The apoptosis of primary osteoblasts was evaluated by Annexin V-FITC/PI Apoptosis Assay Kit (KeyGen Biotech, China). Briefly, primary osteoblasts were collected and washed with PBS. A total of 500 *μ*l binding buffer was added to suspend cells. Firstly, 5 *μ*l Annexin V-FITC was added, and then, 5 *μ*l propidium iodide was added for incubation for 5-15 min in the dark at room temperature. Cell apoptosis was analyzed by flow cytometry (Becton-Dickinson, FACSCalibur, USA).

### 2.9. Western Blot Analysis

The total protein of primary osteoblasts was extracted using RIPA lysis buffer (Beyotime, China) and quantified by BCA assay (Beyotime, China). Equal amounts of proteins (100 *μ*g) were separated via BeyoGel™ Plus PAGE (Beyotime, China) and then transferred to a PVDF membrane (Millipore, USA). After transferring, the membranes were blocked with 5% fat-free milk for 1 h. The membranes were incubated with primary antibodies (Bax, ab32503; Caspase-3, ab32351; and cleaved-Caspase-3, ab32042) purchased from Abcam (USA) and GAPDH, 10494-I-AP purchased from Proteintech (China) at 4°C overnight. The membranes were incubated with second antibodies (goat anti rabbit IgG HRP SE134, goat anti mouse IgG HRP SE131, Solarbio, China) at 37°C for 1 h. The ECL system (CLINX, China) was used for exposing protein bands. The intensity of the bands was analyzed using Image Lab (version 3.0, Bio-Rad, USA).

### 2.10. Gene Expression Analysis Using Quantitative Real-Time Polymerase Chain Reaction (qRT-PCR)

Total RNA was extracted from cells using TRIzol reagent (Invitrogen, USA) according to the manufacturer's protocol. For detecting miR-141-5p expression, MicroRNA cDNA Synthesis Kit (Vazyme, China) was applied for reverse transcription of cDNA, followed by qRT-PCR analysis. U6 was employed as the loading control. The primers of miR-141-5p and U6 were purchased from GeneCopoeia (Guangzhou, China). Meanwhile, the expression of lncRNA-KCNQ1OT1 was evaluated by PrimeScript™ Master Mix (Takara, Japan) and GAPDH was employed as the loading control. The data was calculated using the 2^−*ΔΔ*Ct^ method. Primers used in this study are shown in Table 1.

### 2.11. Dual-Luciferase Reporter Assay

The wild-type (WT) sequence of lncRNA-KCNQ1OT1 containing the miR-141-5p binding sites (KCNQ1OT1-WT) and the mutant sequence (KCNQ1OT1-MUT) were cloned into pMIR vectors (Promega, USA), respectively. Primary osteoblasts and HEK293 cells were cotransfected with miR-141-5p or miR-NC and KCNQ1OT1-WT or KCNQ1OT1-MUT. The luciferase activity was detected using Dual-Luciferase Reporter Assay System (Promega, USA) in the dark.

### 2.12. Statistical Analysis

Statistical analysis was performed with SPSS 20.0 software (IBM, Armonk, NY, USA). All quantitative data were described as mean ± SD. One-way ANOVA was used to analyze the statistical differences among three or more groups while unpaired Student's *t*-test was applied to analyze the statistical differences between two groups. *P* < 0.05 was considered statistically significant.

## 3. Results

### 3.1. TNF-*α* Increases miR-141-5p Expression, Suppresses the Viability, and Promotes the Apoptosis of Primary Osteoblasts in a Dose-Dependent Manner

**Figure 1:**
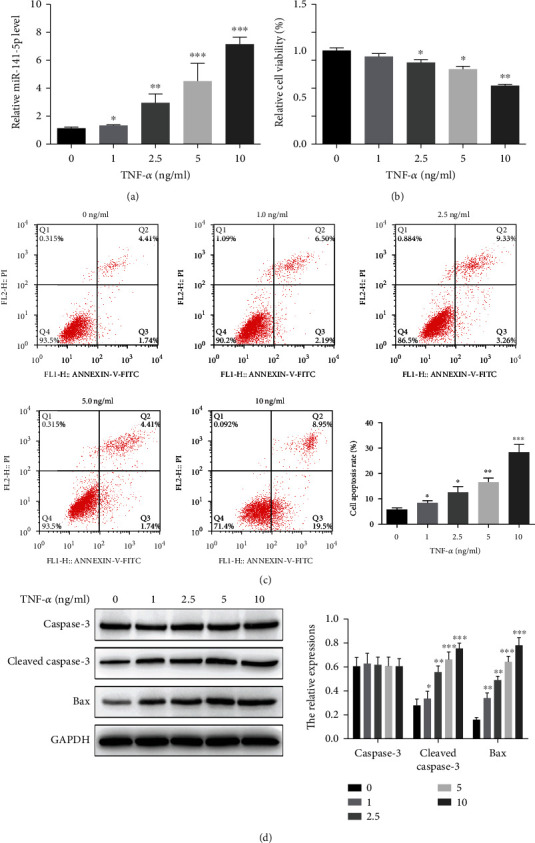
TNF-*α* increases miR-141-5p expression, suppresses the viability, and promotes the apoptosis of primary osteoblasts in a dose-dependent manner. (a) TNF-*α* dose-dependently increased miR-141-5p expression as detected by qRT-PCR. (b, c) TNF-*α* reduced cell viability and increased cell apoptosis in a dose-dependent manner as detected by CCK-8 assay and flow cytometry, respectively. (d) TNF-*α* increased the protein expression level of Bax and cleaved-Caspase-3 dose-dependently as detected by immunoblotting. ^∗^*P* < 0.05, ^∗∗^*P* < 0.01, and ^∗∗∗^*P* < 0.001.

Primary osteoblasts were treated with increased concentrations of TNF-*α* (0, 1, 2.5, 5, and 10 ng/ml). TNF-*α* dose-dependently increased miR-141-5p expression (Figure 1(a)). Meanwhile, primary osteoblasts treated with TNF-*α* showed reduced cell viability and increased apoptosis in a dose-dependent manner (Figures 1(b) and 1(c)). Furthermore, TNF-*α* increased the protein expression level of Bax and cleaved-Caspase-3 dose-dependently (Figure 1(d)).

### 3.2. Knockdown of miR-141-5p Reverses the Effect of TNF-*α* on Primary Osteoblasts

**Figure 2:**
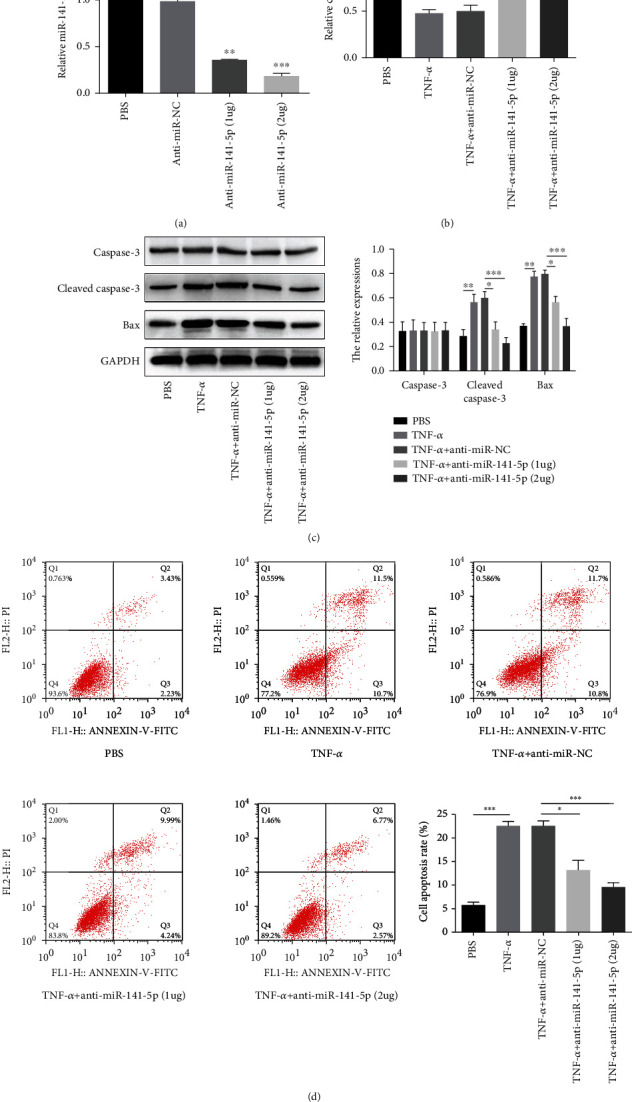
Knockdown of miR-141-5p reverses the effect of TNF-*α* on primary osteoblasts. (a) anti-miR-141-5p reduced the expression of miR-141-5p more significantly in primary osteoblasts compared to anti-miR-NC. (b) CCK-8 assays revealed that the knockdown of miR-141-5p partly reversed the inhibition of cell viability induced by TNF-*α* when cells were treated with PBS, TNF-*α*, TNF-*α*+anti-miR-NC, TNF-*α*+anti-miR-141-5p (1*μ*g), or TNF-*α*+anti-miR-141-5p (2 *μ*g), respectively. (c) The increase of Bax and cleaved-Caspase-3 expression induced by TNF-*α* was inhibited by downregulating miR-141-5p. (d) Annexin V-FITC/PI revealed that the knockdown of miR-141-5p partly reversed cell apoptosis induced by TNF-*α*. ^∗^*P* < 0.05, ^∗∗^*P* < 0.01, and ^∗∗∗^*P* < 0.001.

Since miR-141-5p was upregulated in TNF-*α*-treated primary osteoblasts, we knocked down miR-141-5p expression using anti-miR-141-5p to explore its role in TNF-*α*-induced cytotoxicity and apoptosis. As expected, anti-miR-141-5p reduced the expression of miR-141-5p more significantly in primary osteoblasts compared to anti-miR-NC (Figure 2(a)). CCK-8 and Annexin V-FITC/PI assays revealed that the knockdown of miR-141-5p partly reversed the inhibition of cell viability and the promotion of cell apoptosis induced by TNF-*α* when cells were treated with PBS, TNF-*α*, TNF-*α*+anti-miR-NC, TNF-*α*+anti-miR-141-5p (1 *μ*g), or TNF-*α*+anti-miR-141-5p (2 *μ*g), respectively (Figures 2(b) and 2(d)). In line with this result, the increase of Bax and cleaved-Caspase-3 expression induced by TNF-*α* was inhibited by downregulating miR-141-5p (Figure 2(c)).

### 3.3. Characteristics of ADSCs, Primary Osteoblasts, and ADSCs-Exos

**Figure 3:**
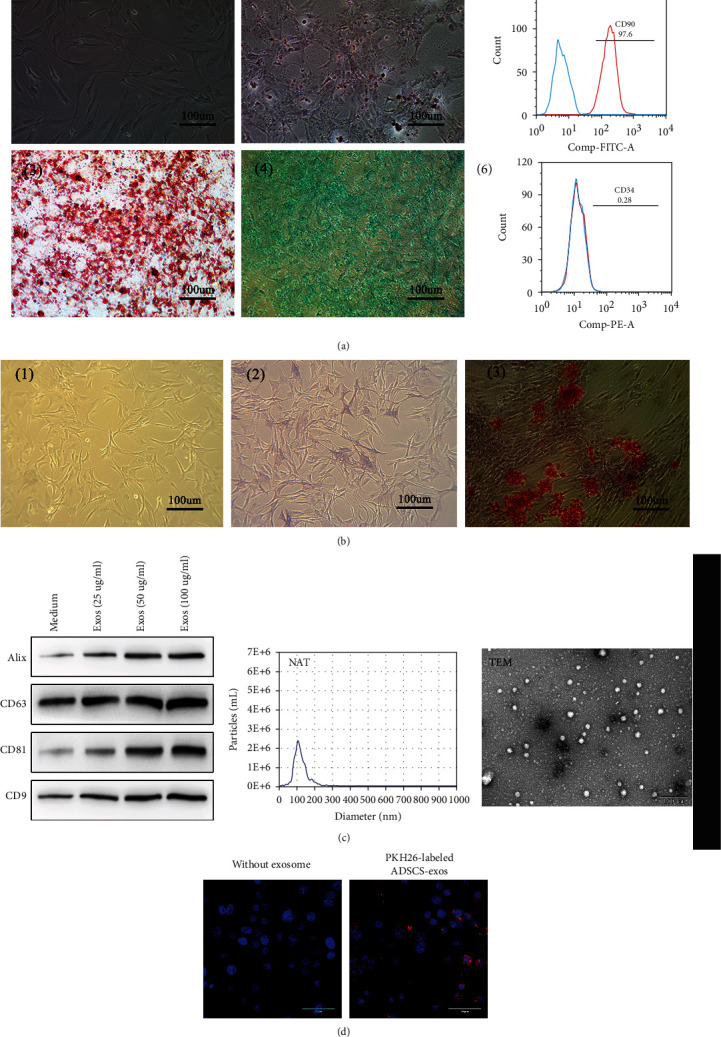
Characteristics of ADSCs, primary osteoblasts, and ADSCs-Exos. (a) (1) ADSCs isolated from C57/BL6 mice had a typical fibroblastic-like morphology. Alizarin Red (2), Oil Red O (3), and Alcian Blue stainings (4) were positive after the induced osteogenic, adipogenic, and chondrogenic differentiations of ADSCs. The isolated ADSCs were positive for CD90 (5) while negative for CD34 (6). (b) (1) Primary osteoblasts isolated from C57/BL6 mice had shuttle, cone, or cube morphology. Alkaline phosphatase staining (2) and Alizarin Red S staining (3) were positive in the isolated osteoblasts. (c) ADSCs-Exos were positive for the exosomes' markers, including CD9, CD81, CD63, and Alix; NAT and TEM results showed that the isolated exosomes were in complete form with the size of 119 + 23.1 nm. (d) Primary osteoblasts could uptake the PKH26-labeled ADSCs-Exos. Red: PKH26-labeled ADSCs-Exos. Blue: nuclei.

**Figure 4:**
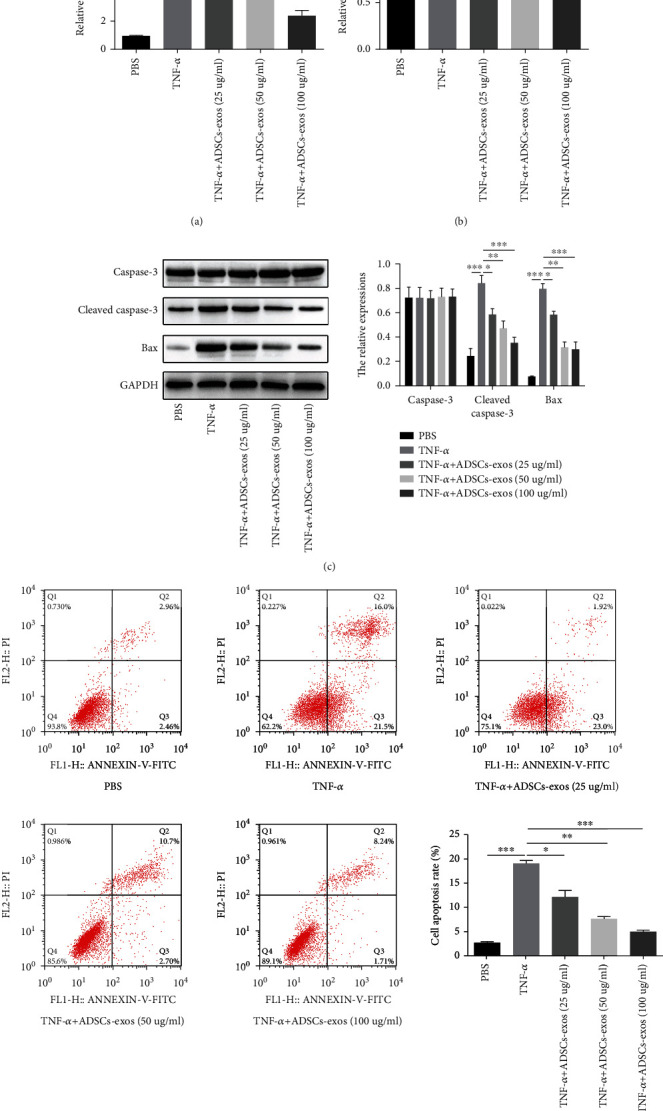
ADSCs-Exos attenuate the effect of TNF-*α* on primary osteoblasts. (a) ADSCs-Exos attenuated the upregulation of miR-141-5p induced by TNF-*α* dose-dependently. (b) ADSCs-Exos mitigated the inhibition of TNF-*α* on cell viability in a dose-dependent manner. (c) ADSCs-Exos dose-dependently decreased the promotion of TNF-*α* on cleaved caspase-3 and Bax expression. (d) Flow cytometry analysis showed that ADSCs-Exos dose-dependently reversed TNF-*α*-induced cell apoptosis. ^∗^*P* < 0.05, ^∗∗^*P* < 0.01, and ^∗∗∗^*P* < 0.001.

**Figure 5:**
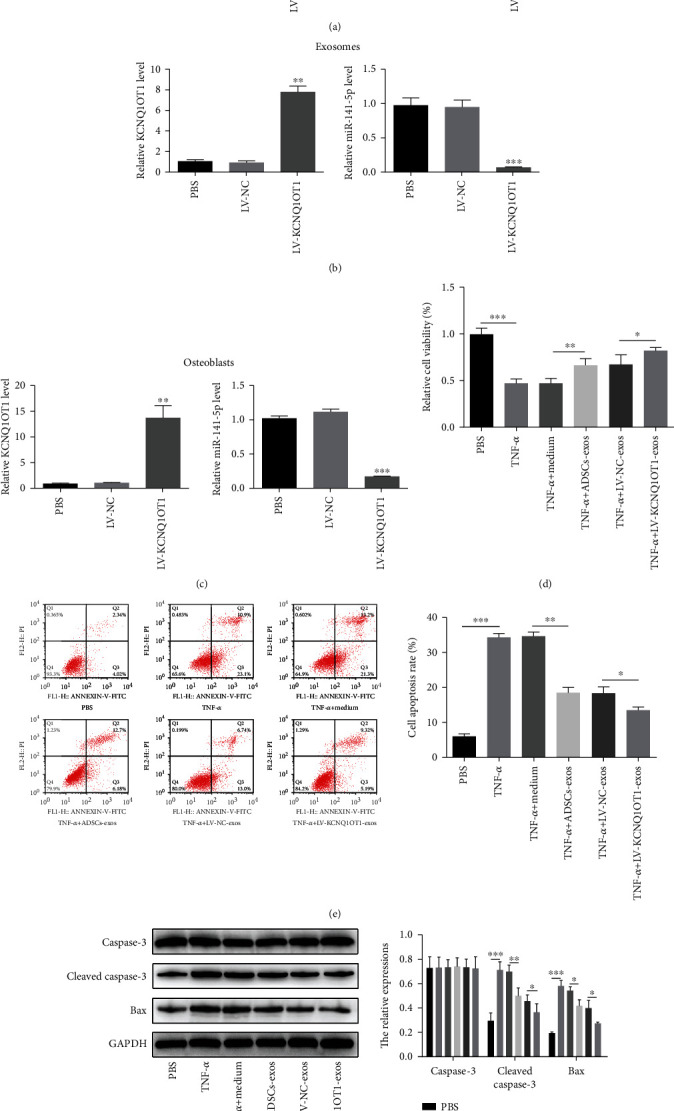
KCNQ1OT1-Exos inhibit TNF-*α*-induced cytotoxicity and apoptosis of primary osteoblasts. (a, b) The expression of KCNQ1OT1 in ADSCs or exosomes derived from the ADSCs after different transfections was elevated, but the expression of miR-141-5p was downregulated compared with that in LV-NC-treated group after the transfection of LV-KCNQ1OT1. (c) KCNQ1OT1 expression was upregulated in primary osteoblasts treated with LV-KCNQ1OT1-Exos compared to NC-Exos. (d) In primary osteoblasts, LV-KCNQ1OT1-Exos mitigated the negative effect of TNF-*α* on cell viability, while ADSCs-Exos exerted a weaker stimulative effect on cell viability compared to LV-KCNQ1OT1-Exos. (e) When primary osteoblasts were cocultured with LV-KCNQ1OT1-Exos, the TNF-*α*-induced cell apoptosis was reversed and ADSCs-Exos exerted a weaker inhibitory effect on cell apoptosis compared to LV-KCNQ1OT1-Exos. (f) The expression of Bax and cleaved caspase-3 in primary osteoblasts was blocked after coculture of LV-KCNQ1OT1-Exos. ^∗^*P* < 0.05, ^∗∗^*P* < 0.01, and ^∗∗∗^*P* < 0.001.

**Figure 6:**
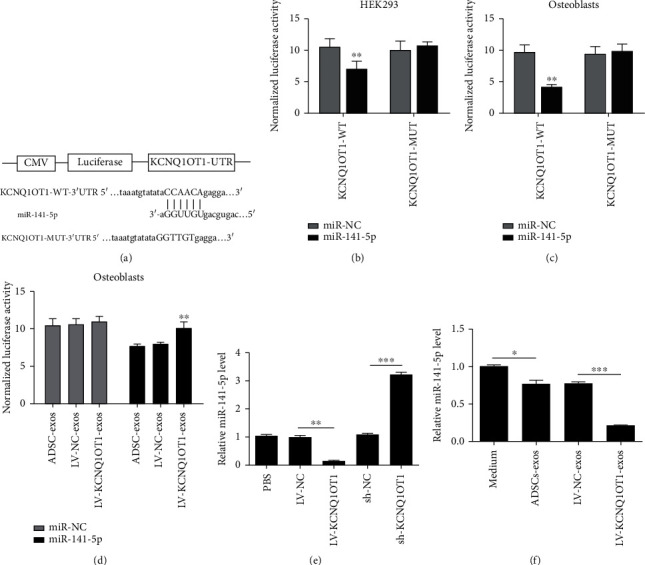
KCNQ1OT1 can sponge miR-141-5p. (a) KCNQ1OT1 and miR-141-5p have potential binding sites. (b, c) As dual-luciferase reporter assay showed, miR-141-5p weakened the luciferase activity in the KCNQ1OT1-WT group obviously, but the activity of luciferase reporters containing KCNQ1OT1-MUT was not changed significantly in both HEK293 and primary osteoblasts. (d) LV-KCNQ1OT1-Exos blocked the inhibitory effect of miR-141-5p on the luciferase activity of reporters containing KCNQ1OT1-WT compared with ADSC-Exos and LV-NC-Exos. (e) KCNQ1OT1 suppressed the expression of miR-141-5p, but the sh-KCNQ1OT1 increased that. (f) The expression of miR-141-5p was downregulated when primary osteoblasts were cocultured with LV-KCNQ1OT1-Exos. ^∗^*P* < 0.05, ^∗∗^*P* < 0.01, and ^∗∗∗^*P* < 0.001.

ADSCs isolated from C57/BL6 mice had a typical fibroblastic-like morphology (Figure 3(a), 1). Alizarin Red (Figure 3(a), 2), Oil Red O (Figure 3(a), 3), and Alcian Blue stainings (Figure 3(a), 4) were positive after the induced osteogenic, adipogenic, and chondrogenic differentiations of ADSCs. The isolated ADSCs were positive for CD90 (Figure 3(a), 5) while negative for CD34 (Figure 3(a), 6). Primary osteoblasts isolated from C57/BL6 mice had shuttle, cone or cube morphology (Figure 3(b), 1). Alkaline phosphatase staining (Figure 3(b), 2) and Alizarin Red S staining (Figure 3(b), 3) were positive in the isolated osteoblasts. The immunoblotting showed that the ADSCs-Exos were positive for the exosomes' markers, including CD9, CD81, CD63, and Alix (Figure 3(c)). NAT and TEM results showed that the isolated exosomes were in complete form with the size of 119 + 23.1 nm (Figure 3(c)). Furthermore, primary osteoblasts were cocultured with PKH26-labeled ADSCs-Exos. The red fluorescence of PKH26 label was observed in primary osteoblasts (Figure 3(d)), indicating primary osteoblasts could uptake ADSCs-Exos.

### 3.4. ADSCs-Exos Attenuate the Effect of TNF-*α* on Primary Osteoblasts

To determine the effects of ADSCs-Exos, primary osteoblasts were treated with TNF-*α* (5 ng/ml) and different doses of ADSCs-Exos (PBS, TNF-*α*, TNF-*α*+ADSCs-Exos (25 *μ*g), TNF-*α*+ADSCs-Exos (50 *μ*g), and TNF-*α*+ADSCs-Exos (100 *μ*g)). ADSCs-Exos attenuated the upregulation of miR-141-5p induced by TNF-*α* dose-dependently (Figure 4(a)). Similarly, ADSCs-Exos reversed the inhibition of cell viability caused by TNF-*α* in a dose-dependent manner (Figure 4(b)). The elevated protein expression of cleaved caspase-3 and Bax induced by TNF-*α* was suppressed after coculture with ADSCs-Exos (Figure 4(c)). In line with this, the results of flow cytometry indicated ADSCs-Exos dose-dependently blocked cell apoptosis induced by TNF-*α* (Figure 4(d)).

### 3.5. KCNQ1OT1-Exos Inhibit TNF-*α*-Induced Cytotoxicity and Apoptosis of Primary Osteoblasts

ADSCs were transfected with LV-KCNQ1OT1 or LV-NC for the determination of whether ADSCs transfected LV-KCNQ1OT1 into secreted exosomes. At 24 h post-LV-KCNQ1OT1 transfection, the expression of KCNQ1OT1 in ADSCs or exosomes derived from the ADSCs after different transfections was elevated, but the expression of miR-141-5p was downregulated compared with that in LV-NC-treated group (Figures 5(a) and 5(b)). In order to confirm whether ADSCs-Exos carrying LV-KCNQ1OT1 could deliver KCNQ1OT1 into primary osteoblasts, primary osteoblasts were cocultured with LV-NC-Exos or LV-KCNQ1OT1-Exos. KCNQ1OT1 expression was upregulated in primary osteoblasts treated with LV-KCNQ1OT1-Exos (Figure 5(c)). Next, to explore whether KCNQ1OT1-Exos could influence TNF-*α*-induced cytotoxicity and apoptosis, primary osteoblasts were treated with TNF-*α* and then cocultured with medium, ADSCs-Exos, LV-NC-Exos, or LV-KCNQ1OT1-Exos. The results of CCK-8 showed the coculture of primary osteoblasts with LV-KCNQ1OT1-Exos mitigated the negative effect of TNF-*α* on cell viability; ADSCs-Exos exerted a weaker stimulative effect on cell viability compared to LV-KCNQ1OT1-Exos (Figure 5(d)). Flow cytometry analysis indicated that TNF-*α*-induced cell apoptosis was reversed when primary osteoblasts were cocultured with LV-KCNQ1OT1-Exos; ADSCs-Exos exerted a weaker inhibitory effect on cell apoptosis compared to LV-KCNQ1OT1-Exos (Figure 5(e)). Consistent with that, the expression of Bax and cleaved caspase-3 in primary osteoblasts was blocked after coculture of LV-KCNQ1OT1-Exos (Figure 5(f)).

### 3.6. KCNQ1OT1 Can Sponge miR-141-5p

Given KCNQ1OT1 sequence contains potential binding sites for miR-141-5p, we predicted KCNQ1OT1 could sponge miR-141-5p (Figure 6(a)). The dual-luciferase reporter assay was conducted to confirm the combination between them. As the result showed, overexpressed miR-141-5p weakened the luciferase activity in the KCNQ1OT1-WT group obviously, but the activity of luciferase reporters containing KCNQ1OT1-MUT was not changed significantly in HEK293 and primary osteoblasts (Figures 6(b) and 6(c)). Compared with ADSC-Exos and LV-NC-Exos, LV-KCNQ1OT1-Exos blocked the inhibitory effect of miR-141-5p on the luciferase activity of reporters containing KCNQ1OT1-WT (Figure 6(d)). Overexpressed KCNQ1OT1 decreased the expression of miR-141-5p, yet downregulation of KCNQ1OT1 increased that (Figure 6(e)). Moreover, when primary osteoblasts were cultured with LV-KCNQ1OT1-Exos, the expression of miR-141-5p was inhibited (Figure 6(f)).

### 3.7. KCNQ1OT1-Exos Inhibit the Effect of TNF-*α* in Primary Osteoblasts by Sponging miR-141-5p

**Figure 7:**
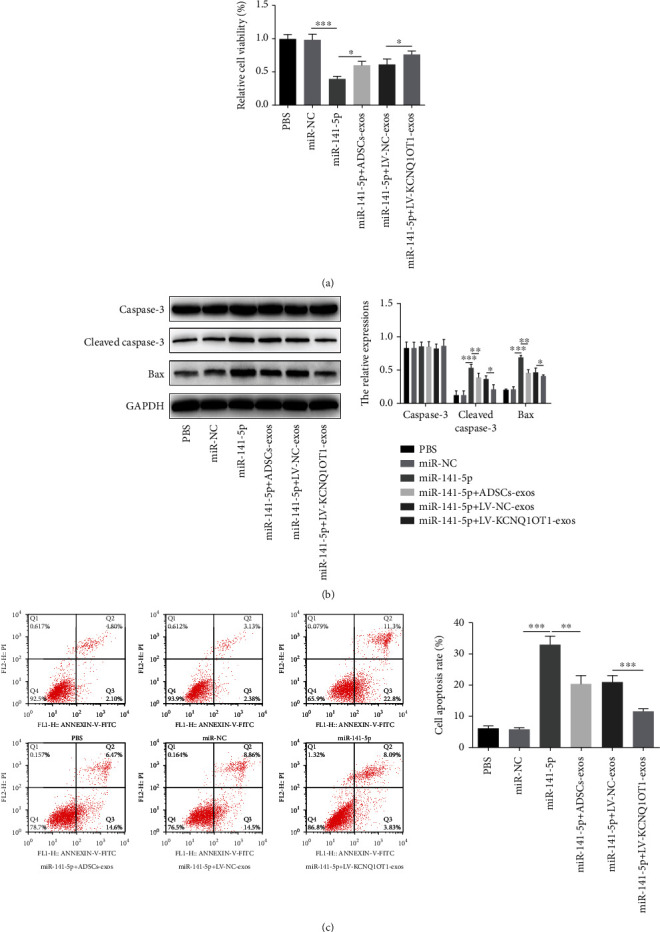
KCNQ1OT1-Exos inhibit the effect of TNF-*α* in primary osteoblasts by sponging miR-141-5p. (a) Primary osteoblasts were treated with TNF-*α* and then treated with miR-NC, miR-141-5p, miR-141-5p+ADSCs-Exos, miR-141-5p+LV-NC-Exos, or miR-141-5p+LV-KCNQ1OT1-Exos, respectively. (b) The inhibitory effect of TNF-*α* on cell viability was blocked by miR-141-5p, and the treatment of ADSCs-Exos or LV-KCNQ1OT1-Exos partly reversed this phenomenon; ADSCs-Exos exerted a weaker stimulative effect on cell viability compared to LV-KCNQ1OT1-Exos; the expression of cleaved caspase-3 and Bax was enhanced by miR-141-5p but was attenuated following coculture with ADSC-Exos or LV-KCNQ1OT1-Exos. (c) As flow cytometry showed, miR-141-5p promoted cell apoptosis while the treatment of ADSC-Exos or LV-KCNQ1OT1-Exos inhibited that; ADSCs-Exos exerted a weaker inhibitory effect on cell apoptosis compared to LV-KCNQ1OT1-Exos. ^∗^*P* < 0.05, ^∗∗^*P* < 0.01, and ^∗∗∗^*P* < 0.001.

Primary osteoblasts were transfected with miR-141-5p or miR-NC to confirm whether KCNQ1OT1-Exos attenuate TNF-*α*-induced cytotoxicity and apoptosis by acting as an miR-146a sponge. After the treatment of TNF-*α*, primary osteoblasts were transfected with miR-141-5p or miR-NC and cocultured with ADSCs-Exos, LV-NC-Exos, or LV-KCNQ1OT1-Exos. The upregulation of miR-141-5p promoted the inhibitory effect of TNF-*α* on cell viability, and coculture with ADSCs-Exos or KCNQ1OT1-Exos partly reversed this phenomenon; ADSCs-Exos exerted a weaker stimulative effect on cell viability compared to LV-KCNQ1OT1-Exos (Figure 7(a)). Similarly, the upregulation of miR-141-5p enhanced cleaved caspase-3 and Bax expression. However, these effects were attenuated following coculture with ADSC-Exos or LV-KCNQ1OT1-Exos (Figure 7(b)). In line with this, flow cytometry showed that overexpressed miR-141-5p promoted cell apoptosis but when cocultured with ADSC-Exos or LV-KCNQ1OT1-Exos, cell apoptosis was inhibited; ADSCs-Exos exerted a weaker inhibitory effect on cell apoptosis compared to LV-KCNQ1OT1-Exos (Figure 7(c)).

## 4. Discussion

With the deepening understanding of osteobiology, skeletal stem cells and osteoblasts are identified as significant targets in the treatment of osteoporosis by promoting bone formation and remodeling [27]. MSC transplantation provides evidences of enhancing osteogenic differentiation, increasing bone mineral density, and halting the deterioration of osteoporosis [28]. In 2001, Zuk et al. [29] isolated ADSCs from adipose tissues and found they are capable of differentiating into adipogenic, osteogenic, chondrogenic, and myogenic cells. As significant bioactive substances released from ADSCs, ADSCs-Exos have exhibited regenerative potential in many diseases [30–32]. However, few studies have investigated the role of ADSCs-Exos in the treatment of osteoporosis. Thus, whether ADSCs-Exos can effectively protect primary osteoblasts from the TNF-*α*-induced cytotoxicity and apoptosis is primarily studied in the present study.

Our results indicate that TNF-*α* can increase miR-141-5p expression and inhibit the cell proliferation in primary osteoblasts. In addition, TNF-*α* can increase cell apoptosis. Consistent with this, the expression of cleaved caspase-3 and Bax was also elevated. Currently, ADSCs have been widely used in tissue regeneration and bioengineering [7, 33]. However, with the in-depth investigations, the application of ADSCs has the potential risk of iatrogenic infection, malignant transformation, immune rejection, and safety issues [34–37]. Compared to ADSCs, ADSCs-Exos can hardly cause the immune rejection and malignant transformation [38, 39]. To make clear the effects of ADSCs-Exos in osteoporosis, we attempt to culture TNF-*α*-treated primary osteoblasts with ADSCs-Exos. Interestingly, ADSCs-Exos promote cell viability and decrease cell apoptosis, suggesting that ADSCs-Exos can be promising candidates in the treatment of osteoporosis.

Although we have found ADSCs-Exos can be beneficial in the treatment of osteoporosis, the underlying mechanism has not been revealed. Exosomes derived from MSCs contain multiple lncRNAs, which can be transported and transferred to other cells to regulate biological functions through targeting downstream genes [40, 41]. KCNQ1OT1, a lncRNA which is closely related to cell proliferation, migration, and apoptosis, has been reported to be an oncogene in a variety of tumors [42]. Evidence has showed that KCNQ1OT1 can promote cell proliferation and migration [43]. Therefore, we are curious whether KCNQ1OT1 can play a positive role in the treatment of osteoporosis. In the present study, KCNQ1OT1-Exos were confirmed to exert a more significant inhibitory effect on TNF-*α*-induced cytotoxicity and apoptosis compared to ADSCs-Exos. Thus, KCNQ1OT1-Exos are expected to be promising candidates in osteoporosis treatment.

lncRNAs can function as miRNA sponges by binding miRNAs [44]. As previously reported, lncRNA-HOTAIR can induce the apoptosis of osteoblasts via modulating the expression of miR-138 [45]. In postmenopausal osteoporosis, lncRNA LOXL1-AS1 regulates osteogenic and adipocytic differentiation of BMSCs via sponging miR-196a-5p [46]. For further mechanistic investigations, we predicted KCNQ1OT1 sequence contained miR-141-5p binding sites *via* bioinformatics analysis. As reported before, miR-141-5p promoted preeclampsia via regulating MAPK1/ERK2 signaling [47]. In chronic myeloid leukemia, microRNA-141-5p acts as a tumor suppressor by downregulating RAB32 [48]. However, the role of miR-141-5p played in osteoporosis has not been reported. In TNF-*α*-treated primary osteoblasts, we found increased expression of miR-141-5p. Moreover, the knockdown of miR-141-5p promoted cell viability and inhibited cell apoptosis induced by TNF-*α*. As dual-luciferase reporter assay showed, miR-141-5p was the target gene of KCNQ1OT1. Furthermore, the rescue experiments revealed that when cocultured with KCNQ1OT1-Exos, the effects induced by miR-141-5p on cell viability and apoptosis in TNF-*α*-treated primary osteoblasts were partly reversed, suggesting that KCNQ1OT1-Exos functioned by sponging miR-141-5p.

## 5. Conclusions

In the present study, we demonstrate that ADSCs-Exos can attenuate cytotoxicity and apoptosis of TNF-*α*-induced primary osteoblasts. KCNQ1OT1-Exos have a more significant inhibitory effect compared to ADSCs-Exos by the function of sponging miR-141-5p, suggesting that KCNQ1OT1-Exos can be promising agents in osteoporosis treatment. Further explorations of the pleiotropic effect of KCNQ1OT1 and the crosstalk between KCNQ1OT1 and miR-141-5p will provide new insights for developing new treatments to improve the therapeutic efficacy based on ADSCs-Exos.

## Figures and Tables

**Table 1:** Sequences of primers used for qRT-PCR.

Primer name	Primer sequence
lncRNA-KCNQ1OT1-F	5′-TTGGTAGGATTTTGTTGAGG-3′
lncRNA-KCNQ1OT1-R	5′-CAACCTTCCCCTACTACC-3′
GAPDH-F	5-TGGATTTGGACGCATTGGTC-3′
GAPDH-R	5′-TTTGCACTGGTACGTGTTGAT-3′
miR-141-5p-F	5′-GCAGTGTTGGATGGTTGAAGTATG-3′
miR-141-5p-R	5′-GAATTTGCGTGTCATCCTTGC-3′
U6-F	5′-CGCTTCGGCAGCACATATACT-3′
U6-R	5′-GAATTTGCGTGTCATCCTTGC-3′

## Data Availability

The data used to support the findings of this study are available from the corresponding authors upon request.
